# Predictors of suboptimal coronary blood flow after primary angioplasty and its implications on short-term outcomes in patients with acute anterior STEMI

**DOI:** 10.1186/s12872-020-01673-0

**Published:** 2020-08-27

**Authors:** Karim Elakabawi, Xin Huang, Sardar Ali Shah, Hameed Ullah, Gary S. Mintz, Zuyi Yuan, Ning Guo

**Affiliations:** 1 Cardiovascular Department, First Affiliated Hospital of Xi’an Jiaotong University, 277 West Yanta Road, Xi’an, 710061 Shaanxi China; 2 Cardiovascular Department, Benha University, Benha, 13518 Egypt; 3 Cardiovascular Research Foundation, New York, NY 10022 USA

**Keywords:** Anterior STEMI, Primary PCI, TIMI flow, Short-term outcomes

## Abstract

**Background:**

Suboptimal coronary blood flow after primary percutaneous coronary intervention (PCI) is a complex multifactorial phenomenon. Although extensively studied, defined modifiable risk factors and efficient management strategy are lacking. This study aims to determine the potential causes of suboptimal flow and associated impact on 30-day outcomes in patients presenting with anterior ST-elevation myocardial infarction (STEMI).

**Methods:**

We evaluated a total of 1104 consecutive patients admitted to our hospital from January 2016 to December 2018 with the diagnosis of anterior wall STEMI who had primary PCI.

**Results:**

Overall, 245 patients (22.2%) had final post-PCI TIMI flow ≤2 in the LAD (suboptimal flow group) and 859 (77.8%) had final TIMI-3 flow (optimal flow group). The independent predictors of suboptimal flow were thrombus burden grade (Odds ratio (OR) 1.848; *p* < 0.001), age (OR 1.039 per 1-year increase; p < 0.001), low systolic blood pressure (OR 1.017 per 1 mmHg decrease; p < 0.001), total stent length (OR 1.021 per 1 mm increase; p < 0.001), and baseline TIMI flow ≤1 (OR 1.674; *p* = 0.018). The 30-day rates of major adverse cardiovascular events (MACE) and cardiac mortality were significantly higher in patients with TIMI flow ≤2 compared to those with TIMI-3 flow (MACE: adjusted risk ratio [RR] 2.021; *P* = 0.025, cardiac mortality: adjusted RR 2.931; *P* = 0.031).

**Conclusion:**

Failure to achieve normal TIMI-3 flow was associated with patient-related (age) and other potentially modifiable risk factors (thrombus burden, admission systolic blood pressure, total stent length, and baseline TIMI flow). The absence of final TIMI-3 flow carried worse short-term clinical outcomes.

## Background

Primary percutaneous coronary intervention (PCI) remains the best option for patients presenting with an acute ST-segment elevation myocardial infarction (STEMI) [1]. While the success rate in the reopening of the thrombotic occlusion can reach up to 95% [2, 3], failure to restore optimal blood flow in the infarct-related coronary artery (i.e., less than thrombolysis in myocardial infarction [TIMI]-3 flow [4]) has been noted in 5–23% of patients and has been associated with adverse clinical outcomes [3, 5, 6]. Many mechanisms have been hypothesized to explain this phenomenon, including the obstruction of epicardial coronary vessels (residual stenosis, thrombus, or dissection) and the disturbances at the level of coronary microcirculation and vascular endothelium (distal embolization of thrombotic materials and plaque debris, leukocyte infiltration, and reperfusion injury) [7, 8]. However, there is a lack of contemporary data on predictors and impacts of suboptimal TIMI flow; and there is still a lack of effective management strategies. We therefore looked at the predictors of suboptimal TIMI flow in patients undergoing primary PCI as well as its impact on 30-day clinical outcomes. Patients with acute anterior STEMI were included to minimize the impact of the left anterior descending (LAD) artery as a strong anatomical risk factor for the development of suboptimal coronary blood flow [9–11].

## Methods

### Study population

This retrospective observational study was performed in the cardiovascular department of First Affiliated Hospital of Xi’an Jiaotong University (Xi’an, China) and included all patients presenting with anterior wall STEMI from January 1, 2016 to December 31, 2018. Included patients were at least 18 years old, presented within 12 h of symptom-onset, and underwent primary PCI. The study was approved by the institutional ethics committee and informed written consent was obtained from all patients.

Anterior STEMI was defined as typical ischemic chest pain lasting ≥20 min with electrocardiographic (ECG) changes (ST-segment elevation ≥1 mm in ≥2 contiguous precordial leads other than V2 and V3 or new-onset left bundle branch block) with a rise in cardiac biomarkers peaking more than twice normal values [12]. The diagnosis was confirmed by coronary angiography showing a LAD and/or left main (LM) lesion. Exclusions included patients with acute MI onset > 12 h or unknown onset, post-CABG patients, or patients who received thrombolytic therapy or platelet glycoprotein IIb/IIIa receptor inhibitors prior to angiography so as not to affect the vessel patency and TIMI flow.

### Study protocol

Medical history, risk factors, physical examination, assessment of vital signs and Killip class, emergency and post-procedural ECG, echocardiography, and full laboratory investigations were obtained together with the related catheterization and PCI data. Information on the clinical outcomes was obtained from outpatient visits, emergency records, or telephone interviews.

Prior to the procedure, 300 mg chewable aspirin were given to the patients along with the recommended dose of ticagrelor or clopidogrel and a weight-adjusted bolus dose of heparin (100 units/kg). Other medications were managed according to the prudence of the doctor in charge and within the scope of local clinical approaches. The radial artery was routinely used in almost all cases. Primary PCI was done according to standard techniques [13]. Implantation of a stent(s) to cover the culprit lesion was routinely implied unless the infarct-related artery (IRA) was heavily calcified or the reference luminal diameter was < 2.5 mm; in these situations, a balloon percutaneous transluminal coronary angioplasty (PTCA) strategy was used. Second-generation drug-eluting stents (DES) were used in all cases with routine pre-dilation and post-dilation optimization. Intravascular ultrasound (IVUS) or optical coherence tomography (OCT) were utilized, if needed, in selected cases.

Two experienced interventional cardiologists evaluated each angiogram with emphasis on (1) the grade of the thrombus burden, (2) the total length of stents used to cover the target lesion, (3) the lesion location (proximal, mid, or distal), (4) the presence of calcification, and (5) the TIMI flow before and after the procedure. Final TIMI blood flow in the LAD artery was determined by two experienced cardiologists as follows: TIMI 0 flow (no perfusion) was the absence of antegrade flow distal to the point of occlusion; TIMI 1 flow (penetration without perfusion) was faint and incomplete perfusion of contrast medium around the thrombus; TIMI 2 flow (partial perfusion) was complete, but delayed perfusion of the distal coronary bed; and TIMI 3 flow (complete perfusion) was an antegrade flow to the entire distal bed at a normal rate [4]. The diagnosis of suboptimal flow was further validated by the corrected TIMI frame count (CTFC) using the LAD artery standard landmark (the most distal branch nearest the apex, commonly referred as the whale’s tail) and a cut-off value of ≤27 frames (quantitative TIMI 3 flow), with patients having CTFC > 27 categorized as having suboptimal flow [14]. *Suboptimal TIMI flow* was TIMI ≤2 flow and/or CTFC > 27 after finishing the procedure, in the absence of dissection, residual stenosis, or spasm [6, 14]. *Thrombus burden* was categorized into 4 grades according to Siano et al.: Grade 0, no thrombus; Grade 1–3, small thrombus burden; and Grade 4, large thrombus burden [15]. *Total ischemic time* was the time from symptom-onset to first balloon inflation.

Patients were subclassified according to the total length of the implanted stent(s) into 3 groups: (short, < 29 mm; medium, 29–50 mm; and long, > 50 mm) and examined for the relation between increasing total stent length and the occurrence of suboptimal flow and cardiac mortality.

### Clinical outcomes

In this study, we compared the effect of the final TIMI flow on the short-term (30-day) clinical outcomes, including differences in cardiovascular mortality, major adverse cardiovascular events (MACE, defined as cardiac death, reinfarction, ischemia-driven target vessel revascularization, or ischemic stroke) and post-catheterization reassessment of general and cardiac status, examining for the development of pump failure, cardiogenic shock, mechanical complication, or malignant arrhythmias between the two study groups. A 12-lead ECG was recorded 45–60 min after the procedure and compared with the admission ECG. The arithmetic sum of ST-segment elevation measured at the J point was calculated from leads V1-V6 (± I, aVL). ST-segment resolution (STR) was classified as complete STR (regression ≥70%) or incomplete STR (unaltered or worsened ST elevation or regression < 70%) [16]. Transthoracic echocardiography was performed within 24 h after primary PCI; the left ventricular ejection fraction (LVEF) and volumes were estimated. The size of infarction was assessed depending on the measure of the peak serum creatine kinase (CK) value, which was obtained from 3 to 5 serial measurements taken routinely within the first 3 days of admission.

*Cardiovascular mortality* was defined as any death related to a proximate cardiac cause including arrhythmia, myocardial ischemia and infarction, pump failure, or cardiac arrest. *Reinfarction* was defined as repeated clinical symptoms or development of new ECG changes associated with a new rise of cardiac troponin (cTn) values >99th percentile upper reference limit (URL) in patients with normal baseline values or an increase of cTn values > 20% of the baseline value when above the 99th percentile URL [12]. *Ischemia-driven target vessel revascularization (TVR)* was defined as repeated revascularization with PCI or coronary artery bypass surgery of the infarct-related artery driven by symptoms or objective evidence of ischemia [13].

### Statistical analysis

Statistical data were presented as means and standard deviations or medians and interquartile ranges (IQR) as appropriate. Categorical data were presented as numbers and percentages. Comparisons between groups (optimal vs. suboptimal flow) were made using the independent t-test or the Mann Whitney U-test for normally and non-normally distributed continuous variables, respectively. Categorical variables were compared using the Chi-square or Fisher’s exact test, as appropriate. A stepwise multivariable logistic regression was used to determine the clinical and angiographic predictors of suboptimal TIMI flow. Variables that showed a marginal association with suboptimal flow on univariable testing (*P* ≤ 0.20) were entered in the multivariable logistic regression model. The variables considered in the model were: age, sex, weight, diabetes mellitus, hypertension, smoking, presenting features (heart rate, systolic and diastolic blood pressures, Killip class, and cardiac arrest), total ischemic time, ticagrelor use, lab findings (serum creatinine, triglycerides, HDL and LDL cholesterol, hemoglobin, high-sensitivity C-reactive protein, and hemoglobinA1c levels), angiographic findings (LM involvement, LAD lesion location, multivessel disease, thrombus burden grade, calcification, baseline TIMI 0/1), number of stents used, total stent length, and mean stent diameter. In the final regression model, only variables with a significant association with suboptimal flow (*P* < 0.05) were included. Odds ratio (OR) and its 95% confidence intervals (CI) were calculated to identify the predictive strength of each variable. Propensity score (PS) matching was applied to identify a cohort of patients with comparable baseline clinical and angiographic features. A propensity score was first estimated for each patient by non-parsimonious multiple logistic regression analysis that included all variables listed in Tables 1 and 2. The matching was then carried out with the use of the single nearest-neighbor matching without replacement, whereby the maximum allowed difference for a match was set at 0.05. This yielded two comparable groups with 214 patients in each group. The PS balance was assessed by comparing the standardized mean differences for the propensity score and the other covariates between the two groups before and after matching. For the measured covariates, an absolute standardized difference < 0.1 indicated a negligible difference between the groups. Analyses in the PS-matched cohort were then performed using a paired t-test or the Wilcoxon signed-rank test for normally and non-normally distributed continuous variables, respectively; and categorical variables were compared using the McNemar’s or Bowker’s test of symmetry, as appropriate. Risk ratio (RR) and accompanying 95% confidence intervals (CI) were calculated to determine the probabilities of 30-days adverse clinical outcomes. Finally, the receiver-operating characteristic (ROC) curve analysis was used to define the best cut-off values for the total stent length consistent with both suboptimal flow and 30-day mortality. All *P*-values were two-sided. P-values ≤0.05 were considered as statistically significant. Data management and statistical analysis were done using SPSS vs.21. (IBM, Armonk, New York, the United States).

**Table 1:** Clinical characteristics of the overall population

Variables	Suboptimal flow (*n* = 245)	Optimal flow (*n* = 859)	*P* value
Mean age (yrs)	62.9 ± 12.8	57.3 ± 12.3	< 0.001
Females, *n (%)*	58 (23.7)	127 (14.8)	0.002
Mean weight (kg)	70.5 ± 11	72.1 ± 12	0.064
Hypertension, *n (%)*	117 (47.8)	390 (45.4)	0.514
Diabetes, *n (%)*	55 (22.4)	161 (18.7)	0.197
Smoking, *n (%)*	114 (46.5)	500 (58.2)	0.001
Previous IHD, *n (%)*	22 (9.0)	72 (8.4)	0.767
Previous CVS, *n (%)*	15 (6.1)	44 (5.1)	0.539
Total ischemic time, hr. Median (IQR)	4.5 (3–8)	5 (3–8)	0.341
Mean admission heart rate, bpm	83.8 ± 13.2	82.5 ± 14.1	0.204
Mean systolic blood pressure, mm Hg	127.5 ± 22.9	135.1 ± 21.3	< 0.001
Mean diastolic blood pressure, mm Hg	82.9 ± 12.8	87.5 ± 13.9	< 0.001
Killip class > 1, *n (%)*	80 (32.7)	163 (19.0)	< 0.001
Cardiac arrest on presentation, *n (%)*	17 (6.9)	31 (3.6)	0.024
Mean serum Creatinine, umol/L	69.12 ± 20.04	65.79 ± 17.74	0.012
Triglyceride, mmol/L, Median (IQR)	1.2 (0.9–1.9)	1.4 (1.0–2.0)	0.067
Mean HDL cholesterol, mmol/L	1.01 ± 0.28	1.03 ± 0.27	0.133
Mean LDL cholesterol, mmol/L	2.55 ± 0.99	2.64 ± 0.93	0.198
Mean Hemoglobin, g/L	142.93 ± 18.4	147.09 ± 17.18	0.001
Mean Platelets count, (×10^9^/L)	213.26 ± 63.53	215.3 ± 65.75	0.665
Mean White blood cells count, (× 10^9^/L)	11.36 ± 3.90	11.09 ± 3.94	0.359
TNT-hs, ng/mL, Median (IQR)	0.4 (0.09–1.5)	0.4 (0.09–1.2)	0.695
Admission CK-MB, U/L, Median (IQR)	42.0 (24.0–134.5)	53(23.0–116.0)	0.992
NT-ProBNP, pg/mL, Median (IQR)	223 (93.0–743.5)	264 (89.0–735.3)	0.774
hsCRP, mg/L, Median (IQR)	3 (1.25–7.3)	2.6 (1.0–6.2)	0.063
Mean HemoglobinA1c%	6.24 ± 1.47	6.09 ± 1.35	0.119

Data are mean ± SD, Median (IQR), or number (%) of patients

*IHD* Ischemic heart disease, *CVS* Cerebrovascular stroke, *HDL* high-density lipoprotein, *LDL* low-density lipoprotein, *CK-MB* Creatine kinase- myocardial band, *TNT-hs* Troponin T-high sensitive, *NT-ProBNP* N-terminal proB-type Natriuretic Peptide, *hsCRP* High-sensitivity C-reactive Protein, *IQR* inter-quartile range

**Table 2:** Angiographic data and primary PCI procedure of the overall population

Variables	Suboptimal flow (*n* = 245)	Optimal flow (*n* = 859)	*P* value
Left main involvement, *n (%)*	29 (11.8)	72 (8.4)	0.098
Number of diseased vessels, *n (%)*			
One	73 (29.8)	263 (30.6)	
Two	97 (39.6)	309 (36.0)	0.555
Three	75 (30.6)	287 (33.4)	
LAD lesion location, *n (%)*			
Proximal	194 (79.2)	624 (72.6)	
Mid	47 (19.2)	226 (26.3)	0.062
Distal	4 (1.6)	9 (1.0)	
Thrombus burden grade 4, *n (%)*	79 (32.2)	123 (14.3)	< 0.001
Moderate/severe calcification, *n (%)*	28 (11.4)	85 (9.9)	0.485
Initial TIMI ≤1, *n (%)*	208 (84.9)	572 (66.6)	< 0.001
Approach, n (%)			
Radial	232 (94.7)	825 (96.0)	0.357
Femoral	13 (5.3)	34 (4.0)	
Stents, *n (%)*			
PTCA	3 (1.2)	23 (2.7)	
One	135 (55.1)	555 (64.6)	0.006
Two	98 (40.0)	266 (31.0)	
Three	9 (3.7)	15 (1.7)	
Total Stent length, mm	40.7 ± 17.2	35.7 ± 14.2	< 0.001
Mean stent diameter, mm	3.02 ± 0.5	2.94 ± 0.6	0.067
Bifurcation stenting, *n (%)*	2 (0.8)	3 (0.3)	0.337
P2Y12 inhibitor, *n (%)*			
Clopidogrel	180 (73.5)	607 (70.7)	0.392
Ticagrelor	65 (26.5)	252 (29.3)	
Thrombus Aspiration, *n (%)*	78 (31.8)	193 (22.5)	0.003
IABP, *n (%)*	37 (15.1)	63 (7.3)	< 0.001

Data are mean ± SD or number (%) of patients

*LAD* Left anterior descending, *TIMI* thrombolysis in myocardial infarction, *PTCA* Percutaneous transluminal coronary angioplasty, *IABP* Intra-aortic balloon pump

## Results

### Clinical characteristics

**Fig. 1:**
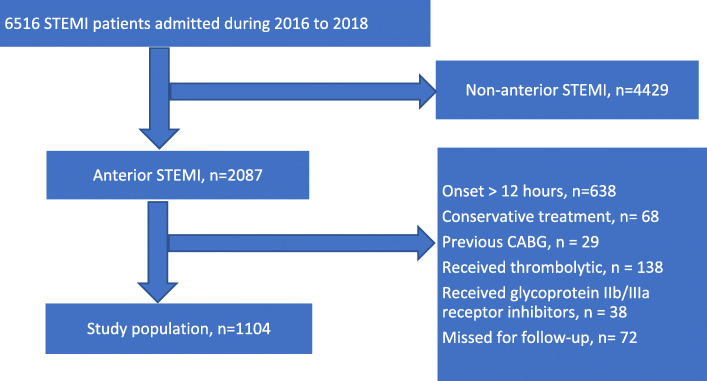
Flowchart of the present study population *STEMI,* ST-elevation myocardial infarction; *CABG*, coronary artery bypass graft

As shown in (Fig. 1), a total of 1104 anterior STEMI patients were enrolled in this study; 245 (22.2%) had final TIMI flow ≤2 after primary PCI (suboptimal group) and 859 (77.8%) patients had final TIMI flow grade 3 (optimal group). The clinical features, risk factors, and laboratory data have been shown in Table 1. Compared to patients with post-PCI TIMI-3 flow, patients with final suboptimal TIMI flow tended to be older with lower systolic and diastolic blood pressure, higher Killip class at admission, and higher prevalence of cardiac arrest in the emergency department. Additionally, female sex, less smoking, higher baseline serum creatinine, and lower hemoglobin levels were significantly associated with the occurrence of suboptimal post-PCI TIMI flow. On the other hand, there were no significant differences in the total ischemic time or other risk factors between the two study groups.

### Angiographic data and primary PCI procedure

As shown in Tables 2 and 3, patients in the suboptimal group had low (≤1) initial TIMI flow, high TIMI thrombus burden grade after wiring, greater need for implantation of ≥1 stent, and longer mean implanted stent length. The glycoprotein IIb/IIIa receptor antagonist tirofiban and thrombus aspiration were used more often in patients with post-PCI TIMI ≤2 flow. The need for hemodynamic support and/or intravenous (IV) infusion of noradrenaline occurred more frequently in the suboptimal group. Intracoronary (IC) arteriolar vasodilators were used in all cases with TIMI ≤2 flow, whether transient or permanent, but were significantly utilized in the suboptimal group. Additionally, there was a non-significant trend in patients with suboptimal TIMI flow to have a proximal LAD or LM lesion location and a larger stent diameter. Conversely, there were no statistical differences in the number of diseased vessels, lesion calcification, or access site selection between the two groups.

**Table 3:** Cath-lab medications and post-procedural clinical parameters

Variables	Total population			Propensity-matched population		
	Suboptimal flow (*n* = 245)	Optimal flow (*n* = 859)	*P* value	Suboptimal flow (*n* = 214)	Optimal flow (*n* = 214)	*P* value
**Medications used during primary PCI**						
Glycoprotein IIb/IIIa inhibitor, *n (%)*	65 (26.5)	99 (11.5)	< 0.001	56 (26.2)	40 (18.7)	0.098
IC Nitroprusside, *n (%)*	37 (15.1)	56 (6.5)	< 0.001	35 (16.4)	5 (2.3)	< 0.001
IC Adrenaline, *n (%)*	23 (9.4)	28 (3.3)	< 0.001	18 (8.4)	11 (5.1)	0.178
Atropine, *n (%)*	27 (11.0)	72 (8.4)	0.202	22 (10.3)	26 (12.1)	0.643
Noradrenaline, *n (%)*	106 (43.3)	128 (14.9)	< 0.001	91 (42.5)	35 (16.4)	< 0.001
**Post-procedural clinical parameters**						
Creatine kinase peak, U/L, Median (IQR)	5268 (3829.0–7422.0)	4215 (2300.0–6409.0)	< 0.001	5265 (3829.0–7422.0)	4542 (2156.3–6740.5)	0.002
ST segment resolution, n (%)						
< 70%	109 (44.5)	315 (36.7)	0.026	97 (45.3)	80 (37.4)	0.108
> 70%	136 (55.5)	544 (63.3)		117 (54.7)	134 (62.6)	
Mean Ejection Fraction (%)	43.8 ± 6.2	46.2 ± 6.9	< 0.001	43.71 ± 6.41	45.34 ± 7.48	0.015
Hospital stay (days), Median (IQR)	4 (3–6)	4 (3–5)	< 0.001	4 (3–6)	4 (3–6)	0.864

Data are mean ± SD, Median (IQR), or number (%) of patients

*IC* intracoronary, *IQR* inter-quartile range

### Baseline clinical and angiographic characteristics of the PS-matched population

After performing PS matching, a total of 214 matched patient pairs were created. In the matched cohort, there were no significant differences in baseline clinical or angiographic characteristics (data are presented in Tables 4 and 5). However, IC sodium nitroprusside and IV infusion of noradrenaline were used more frequently in the suboptimal group (Table 3).

**Table 4:** Clinical characteristics of the propensity-matched population

Variables	Suboptimal flow (*n* = 214)	Optimal flow (n = 214)	*P* value
Mean age (yrs)	61.75 ± 12.8	63.60 ± 12.6	0.131
Females, *n (%)*	48 (22.4)	58 (27.6)	0.229
Mean weight (kg)	70.50 ± 12.20	68.72 ± 11.7	0.127
Hypertension, *n (%)*	103 (48.1)	114 (53.3)	0.338
Diabetes, *n (%)*	43 (20.1)	40 (18.7)	0.801
Smoking, *n (%)*	102 (47.7)	106(49.5)	0.769
Previous IHD, *n (%)*	20 (9.3)	30 (14)	0.175
Previous CVS, *n (%)*	14 (6.5)	16 (7.5)	0.855
Total ischemic time, hr. Median (IQR)	5 (3–8)	4 (3–7)	0.548
Mean admission heart rate, bpm	83.96 ± 13.3	82.44 ± 14.8	0.252
Mean systolic blood pressure, mm Hg	129.17 ± 22.6	131.54 ± 21.5	0.232
Mean diastolic blood pressure, mm Hg	83.57 ± 12.9	83.85 ± 13.2	0.82
Killip class > 1, *n (%)*	66 (30.8)	57 (26.6)	0.380
Cardiac arrest on presentation, *n (%)*	12 (5.6)	13 (6.1)	0.837
Mean serum Creatinine, umol/L	68.56 ± 19.5	67.81 ± 19.1	0.68
Triglyceride, mmol/L, Median (IQR)	1.27 (0.93–1.9)	1.20 (0.90–1.9)	0.622
Mean HDL cholesterol, mmol/L	1.01 ± 0.28	0.97 ± 0.29	0.170
Mean LDL cholesterol, mmol/L	2.60 ± 1.02	2.46 ± 0.91	0.122
Mean Hemoglobin, g/L	144.29 ± 18.07	142.06 ± 18.35	0.171
Mean Platelets count, (×10^9^/L)	213.68 ± 60.59	214.96 ± 70.24	0.840
Mean White blood cells count, (×10^9^/L)	11.30 ± 3.92	11.46 ± 4.24	0.690
TNT-hs, ng/mL, Median (IQR)	0.4 (0.1–1.5)	0.33 (0.08–1.085)	0.150
Admission CK-MB, U/L, Median (IQR)	41.5 (25.75–131.5)	48.0(21–102.5)	0.492
NT-ProBNP, pg/mL, Median (IQR)	223 (93.75–722.5)	250 (90.75–847.5)	0.061
hsCRP, mg/L, Median (IQR)	2.9 (1.2–6.88)	3.3 (1.1–8.5)	0.396
Mean HemoglobinA1c %	6.11 ± 1.31	5.99 ± 1.1	0.270

Data are mean ± SD, Median (IQR), or number (%) of patients

*IHD* Ischemic heart disease, *CVS* Cerebrovascular stroke, *HDL* high-density lipoprotein, *LDL* low-density lipoprotein, *CK-MB* Creatine kinase- myocardial band, *TNT-hs* Troponin T-high sensitive, *NT-ProBNP* N-terminal proB-type Natriuretic Peptide, *hsCRP* High-sensitivity C-reactive Protein, *IQR* inter-quartile range

**Table 5:** Angiographic data and primary PCI procedure of the propensity-matched population

Variables	Suboptimal flow (*n* = 214)	Optimal flow (*n* = 214)	*P* value
Left main involvement, *n (%)*	24 (11.2)	15 (7)	0.188
Number of diseased vessels, *n (%)*			
One	68 (31.8)	73 (34.1)	
Two	79 (36.9)	71 (33.2)	0.865
Three	67 (31.3)	70 (32.7)	
LAD lesion location, *n (%)*			
Proximal	166 (77.6)	178 (83.2)	
Mid	44 (20.6)	36 (16.8)	0.082
Distal	4 (1.9)	0 (0)	
Thrombus burden grade 4, *n (%)*	60 (28)	60 (28)	1.0
Moderate/severe calcification, *n (%)*	22 (10.3)	33 (15.4)	0.161
Initial TIMI ≤1, *n (%)*	178 (83.2)	165 (77.1)	0.118
Approach, n (%)			
Radial	207 (96.7)	203 (94.9)	0.335
Femoral	7 (3.3)	11 (5.1)	
Stents, *n (%)*			
PTCA	0 (0)	0 (0)	
One	123 (57.5)	133 (62.1)	0.241
Two	83 (38.8)	76 (35.5)	
Three	8 (3.7)	5 (2.3)	
Total Stent length, mm	39.99 ± 17.28	37.53 ± 14.97	0.106
Mean stent diameter, mm	3.03 ± 0.36	3.06 ± 0.37	0.512
Bifurcation stenting, *n (%)*	2 (0.9)	2 (0.9)	1.0
P2Y12 inhibitor, *n (%)*			
Clopidogrel	154 (72.0)	153 (71.5)	0.915
Ticagrelor	60 (28.0)	61 (28.5)	
Thrombus Aspiration, *n (%)*	64 (29.9)	77 (36.0)	0.208
IABP, *n (%)*	24 (11.2)	20 (9.3)	0.635

Data are mean ± SD or number (%) of patients

*LAD* Left anterior descending, *TIMI* thrombolysis in myocardial infarction, *PTCA* Percutaneous transluminal coronary angioplasty, *IABP* Intra-aortic balloon pump

### Independent predictors of suboptimal flow

Multivariable stepwise logistic regression analysis identified thrombus burden grade (OR 1.848; 95% CI 1.532–2.229, *p* < 0.001), older patient age (OR 1.039 per 1-year increase; 95% CI 1.026–1.052, p < 0.001), lower systolic blood pressure (OR 1.017 per 1 mmHg decrease; 95% CI 1.009–1.025, *p* < 0.001), increased total stent length (OR 1.021 per 1 mm increase; 95% CI 1.011–1.032, p < 0.001), and baseline TIMI flow ≤1 (OR 1.674; 95% CI 1.094–2.562, *p* = 0.018) as independent predictors of suboptimal flow after primary PCI in patients with anterior STEMI (Table 6).

**Table 6:** Multivariable logistic regression analysis of predictors of suboptimal flow after primary PCI

Variables	OR	95% CI for OR	*P* value
Thrombus burden, grade	1.848	1.532–2.229	< 0.001
Age, per 1-year increase	1. 039	1.026–1.052	< 0.001
Systolic blood pressure, per 1 mmHg decrease	1.017	1.009–1.025	< 0.001
Total stent length, per 1 mm increase	1.021	1.011–1.032	< 0.001
Baseline TIMI flow ≤1	1.674	1.094–2.562	0.018

*OR* Odds ratio, *95% CI* 95% Confidence interval

*TIMI* thrombolysis in myocardial infarction

### 30-day clinical outcomes

The suboptimal group had higher peak CK value, worse LVEF after PCI, lower rates of complete ST-segment resolution, and more prolonged hospital stay than the optimal group (Table 3).

**Fig. 2:**
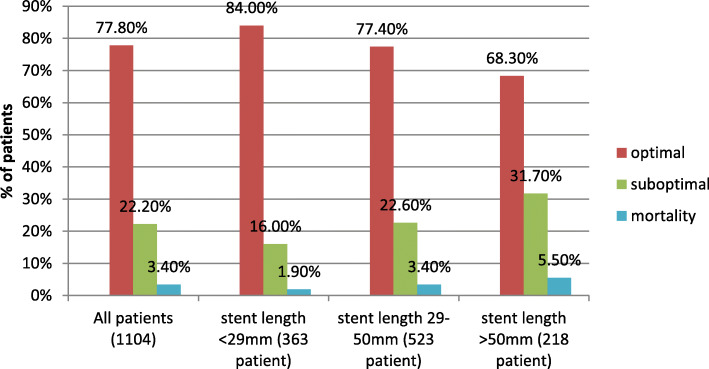
Relationship of total deployed stent(s) length with rates of final suboptimal flow and 30-day cardiac mortality

The 30-day clinical outcomes of the total population have been shown in Table 7. Patients with final TIMI flow ≤2 had more 30-day MACE (13.9% vs. 6.1%; unadjusted RR 2.416, 95% confidence interval [CI] 1.522–3.833, p < 0.001), cardiac death (7.3% vs. 2.2%; unadjusted RR 3.506, 95% CI 1.810–6.790, p < 0.001), and a higher 30-day incidence of myocardial infarction (7.3% vs. 3.5%; unadjusted RR 2.118, 95% CI 1.163–3.856, *p* = 0.014) and need for repeat revascularization (6.1% vs. 3.3%; unadjusted RR 1.936, 95% CI 1.017–3.685, *p* = 0.044). In sub-group analysis, it was observed that patients with longer total implanted stent length had significantly higher rates of both final suboptimal flow (*P* < 0.001) and 30-day cardiac mortality (*P* = 0.021), (Fig. 2).

**Table 7:** 30-day clinical outcomes

Variables	Total population				Propensity-matched population			
	Suboptimal flow (*n* = 245)	Optimal flow (*n* = 859)	Unadjusted RR (95% CI)	*P* value	Suboptimal flow (n = 214)	Optimal flow (n = 214)	Adjusted RR (95% CI)	*P* value
MACE, n (%)	34 (13.9)	52 (6.1)	2.416 (1.522–3.833)	< 0.001	29 (13.6)	14 (6.5)	2.021 (1.097–3.535)	0.025
Mortality (cardiac), n (%)	18 (7.3)	19 (2.2)	3.506 (1.810–6.790)	< 0.001	15 (7.0)	5 (2.3)	2.931 (1.073–7.442)	0.031
Re-infarction, n (%)	18 (7.3)	30 (3.5)	2.118 (1.163–3.856)	0.014	17 (7.9)	8 (3.7)	2.060 (0.905–4.438)	0.108
Revascularization, n (%)	15 (6.1)	28 (3.3)	1.936 (1.017–3.685)	0.044	14 (6.5)	6 (2.8)	2.271 (0.889–5.465)	0.115
Ischemic stroke, n (%)	1 (0.4)	3 (0.3)	1.169 (0.122–11.185)	0.892	1 (0.5)	0 (0)	NA	0.995
Cardiogenic shock, n (%)	33 (13.5)	45 (5.2)	2.829 (1.761–4.545)	< 0.001	25 (11.7)	12 (5.6)	2.061 (1.040–3.852)	0.039
Heart Failure, n (%)	84 (34.3)	164 (19.1)	2.211 (1.615–3.026)	< 0.001	69 (32.2)	48 (22.4)	1.412 (1.024–1.866)	0.036
Malignant arrhythmias, n (%)	42 (17.1)	88 (10.2)	1.813 (1.217–2.701)	0.003	37 (17.3)	20 (9.3)	1.850 (1.119–2.914)	0.017
Bradyarrhythmias, n (%)	27 (11.0)	65 (7.6)	1.513 (0.943–2.428)	0.084	22 (10.3)	19 (8.9)	1.143 (0.629–1.997)	0.655
Bleeding, n (%)	14 (5.7)	34 (4.0)	1.471 (0.776–2.787)	0.237	10 (4.7)	7 (3.3)	1.360 (0.517–3.417)	0.529

Data are number (%) of patients

*RR* Risk ratio, *95% CI* 95% Confidence interval, *MACE* Major adverse cardiovascular events (cardiac death, reinfarction, ischemia-driven target vessel revascularization, or ischemic stroke)

In the PS-matched cohort, the overall findings were similar. Patients in the suboptimal group had higher peak CK value and worse LVEF post-PCI (Table 3). Cumulative rates of 30-day MACE occurred in 13.6% of anterior STEMI patients with suboptimal TIMI flow vs 6.5% of patients with final TIMI flow 3 (adjusted RR 2.021, 95% CI 1.097–3.535, *P* = 0.025). Cardiac mortality was higher in STEMI patients with suboptimal TIMI flow (7.0% vs. 2.3%, adjusted RR 2.931, 95% CI 1.073–7.442, *P* = 0.031). However, there were no significant differences in myocardial infarction (*P* = 0.108), TVR (*P* = 0.115), ST-segment resolution (P = 0.108), or total hospital stay (*P* = 0.864) between the two groups (Tables 3 and 7).

## Discussion

The present study identified certain independent predictors of suboptimal TIMI flow and evaluated short-term outcomes of 1104 anterior STEMI patients treated with primary PCI at a median of 5 h after symptom-onset.

The main features of the study can be summarized as follows:

(1) Despite the constant development of PCI techniques, pharmacotherapeutics and interventional devices in the current era, failure to achieve normal TIMI flow occurred in almost one-quarter of anterior STEMI patients and was associated with patient-specific factors (advanced age and low admission systolic blood pressure) and angiographic/procedural factors (baseline TIMI ≤1flow, heavy thrombus burden, and increased total deployed stent length). (2) The 30-day cardiac mortality and MACE rates were significantly greater in patients with TIMI flow ≤2 versus TIMI-3 flow as well as a lower LVEF; increased total CK (indicating larger infarct size); and more frequent heart failure, cardiogenic shock, and malignant arrhythmias.

This study is unique in that it included only patients with acute anterior STEMI having the LAD as the culprit artery and who were presented within 12 h of symptom-onset. Strong points of this study are the relatively large number of patients and the implementation of modern interventional and therapeutic strategies known to improve the outcomes of STEMI patients, including 2nd generations DES, non-compliant (NC) balloon post-dilation, dual antiplatelet therapy, availability of intracoronary imaging, and minimization of the door-to-balloon time. Previous studies were mainly conducted in the era of bare-metal stents or 1st generations DES and included all STEMI patients (not just anterior STEMI) with a time interval extended up to 24 h [9–11, 17]. This may in part explain the relatively high rate of suboptimal flow observed in our study (22.2%) compared with previously published rates (5–23%) [3, 5]. Many studies have illustrated the relationship between larger infarct size and the increased prevalence of suboptimal flow [11, 18]; an IRA lesion in the proximal LAD typically accounts for the largest myocardial infarctions [18].

### Clinical and angiographic predictors of suboptimal flow

In our study, older patients and those with lower blood pressure had higher rates of suboptimal flow post-PCI. Older patients have more diffuse atherosclerosis, increased calcification, and more distal microembolization [17]. Besides, earlier studies noted that advanced age, LVEF < 50%, increased heart rate, and lower systolic blood pressure (< 90 mmHg) were predictors of suboptimal TIMI flow and higher in-hospital mortality [19–21].

Primary PCI in the setting of a ruptured plaque with thrombus and atherosclerotic debris leads to distal embolization of microthrombi and plaque contents [22]. This distal microembolization results in increased distal resistance, multiple microinfarcts, and more myocardial necrosis leading to a reduction of coronary blood flow [23]. In our study, heavy thrombus burden was a powerful independent predictor of suboptimal coronary blood flow post-primary PCI. Conversely, De Luca et al. noted that suboptimal coronary flow can happen even in acute STEMI with minimal thrombus burden, but with longer reperfusion times. Prolonged ischemia can adversely affect the microvascular circulation leading to the development of suboptimal flow [24]. However, in our study, there was no correlation between total ischemic time and final TIMI flow, inconsistent with some [10, 17–19], but not all previously published studies [9, 25].

The mean total stent length in the present study was 36.8 mm, noticeably longer than most previous studies [9, 17]. In addition, we performed routine balloon pre-dilation and NC balloon post-dilation after stent deployment to achieve optimum stent expansion and angiographic results. This increasingly used PCI strategy may also help to explain the higher prevalence of suboptimal flow in our contemporary practice. Longer stents and the use of pre- and post-dilation increase the likelihood of mechanical fragmentation with subsequent dislodgement of thrombus and plaque components [26].

In the present study, baseline TIMI flow ≤1 was associated with a final suboptimal flow. This finding is reinforced by the findings of preceding studies which clarified that early and adequately patent IRA is associated with smaller infarct size and enhanced endogenous lysis of thrombotic material meaning lower thrombotic burden and decreased incidence of coronary spasm, distal microembolization and microvascular disruption which are the main mechanisms for suboptimal TIMI flow [9, 10, 24].

### Modifiable risk factors and clinical implications

Our findings together with previously published reports emphasize suboptimal coronary blood flow after primary PCI as a main indicator of adverse outcomes. Therefore, early recognition and control of risk factors and proper management of suboptimal TIMI flow are extremely necessary [27]. In the present study, we identified heavy thrombus burden, low admission systolic blood pressure, use of longer stents, and baseline TIMI flow ≤1 as significant and independent risk factors of developing suboptimal flow.

Properly managed heavy thrombus burden with liberal use of Glycoprotein IIb/IIIa receptor antagonists, aspiration catheters, distal protection devices or mesh-covered embolic protection stents, especially in the presence of other risk factors such as old age and a long target lesion, may improve distal TIMI flow and outcomes in the high-risk patients [28, 29]. An alternative strategy suggested by Isaaz et al. is to give glycoprotein IIb/IIIa inhibitors or low dose thrombolytic therapy and delay stenting if a satisfactory antegrade flow is obtained by pre-dilation alone [30].

Longer stent length has been described to be associated with worse outcomes [31], but little is known about the impact of DES length on suboptimal flow and outcomes in patients with acute STEMI. We categorized patients according to the length of the deployed stent(s) vs suboptimal flow and mortality (Fig. 2). We noted that the rates of suboptimal flow almost doubled (16.0% vs. 31.7%, *P* < 0.001) and mortality rates tripled (1.9% vs. 5.5%; *p* = 0.019) in patients with longer (total stent length > 50 mm) stents compared to patients with stent length < 29 mm. This suggests an approach of covering the culprit lesion with the shortest stent possible, avoiding unnecessary balloon dilations, and use direct or deferred stenting if feasible [17].

Patients presenting with hypotension and cardiogenic shock are more likely to experience suboptimal flow. Pharmacological management of suboptimal flow in these cases may be challenging, as most of the agents used to reverse the suboptimal flow are vasodilatory and may cause further hypotension. Adequate management of hypotension with vasopressors, IABP, or other hemodynamic support devices in high-risk patients may help to maintain adequate coronary perfusion pressure and overcome microvascular obstruction to improve distal TIMI flow and primary PCI outcomes [19, 20]. Similarly, restoring patency of the IRA as soon as possible is strongly linked to better outcomes [9, 10]. Despite being the best option to achieve IRA patency, primary PCI may not be readily available in all institutions or all geographical regions; in that case the administration of glycoprotein IIb/IIIa inhibitors or thrombolytic therapy may be considered to achieve early patency of the IRA when primary PCI is not available or the transfer time is presumed to be prolonged.

There is currently no effective treatment for suboptimal flow, and it is still safer to try to avoid the development of suboptimal flow than to manage it when it is established. Measures should be implied to increase public awareness of the symptoms of heart attacks and the importance of seeking immediate medical care. In addition, efforts to minimize the door-to-balloon time in conjunction with improvements in adjunctive pharmacotherapeutics and interventional techniques are urgently required to help the prevention of this abnormality.

### Study limitations

There are several potential limitations to the current study. First, it is a single-center retrospective study. The results are therefore affected by some confounding factors and possible selection bias. Propensity matching and appropriate statistical adjustments were performed to control this limitation, but the influence of some unmeasured confounders cannot be eliminated completely. Second, patients who received thrombolytic therapy or platelet glycoprotein IIb/IIIa receptor inhibitors prior to angiography were excluded. In addition, at the time the study was performed, novel anti-platelets (ticagrelor and prasugrel) were administered to a relatively small number of patients, the use of such therapies could have an impact on thrombotic events and would have decreased the incidence of suboptimal flow. Third, we did not assess myocardial blush. Fourth, we did not evaluate microvascular obstruction using specific diagnostic techniques such as cardiac magnetic resonance imaging, contrast echocardiography, or nuclear imaging. Fifth, we did not perform direct stenting; and we used routine balloon pre- and post-dilatation in almost all cases. Finally, long-term follow up was not available.

## Conclusions

Suboptimal TIMI flow is the last barrier before the achievement of successful reperfusion after primary PCI. In the present study, failure to restore normal TIMI-3 flow occurred in 22.2% of patients presenting with anterior STEMI and was associated with patient-related (advanced age) and other potentially modifiable risk factors (thrombus burden grade, admission systolic blood pressure, total stent length, and baseline TIMI flow). Absence of final TIMI-3 flow after primary PCI carried worse short-term outcomes and increased 30-day mortality compared to patients in whom TIMI flow was normalized by primary PCI.

## Data Availability

The data used to support the findings of this study are available from the First Affiliated Hospital of Xi’an Jiaotong University, the data are available from the authors upon reasonable request and with permission from the Hospital.

## Abbreviations

PCI: Percutaneous coronary intervention
STEMI: ST-elevation myocardial infarction
LAD: Left anterior descending
LM: Left main
CTFC: Corrected TIMI frame count
IRA: Infarct-related artery
PTCA: Percutaneous transluminal coronary angioplasty
DES: Drug-eluting stents
MACE: Major adverse cardiovascular events
TVR: Target vessel revascularization
STR: ST-segment resolution
URL: Upper reference limit
LVEF: Left ventricular ejection fraction
CK: Creatine kinase
PS: Propensity score
OR: Odds ratio
CI: Confidence interval
RR: Risk ratio
NC: Non-compliant

## References

[1] Guarini G, Huqi A, Morrone D, Capozza P, Todiere G, Marzilli M (2014). Pharmacological approaches to coronary microvascular dysfunction. Pharmacol Ther.

[2] Gibson CM, Schömig A (2004). Coronary and myocardial angiography: angiographic assessment of both epicardial and myocardial perfusion. Circulation..

[3] Rezkalla SH, Kloner RA (2008). Coronary no-reflow phenomenon: from the experimental laboratory to the cardiac catheterization laboratory. Catheter Cardiovasc Interv.

[4] The Thrombolysis in Myocardial Infarction (TIMI) Trial: Phase I Findings. N Engl J Med. 1985;312:932–6. doi:10.1056/NEJM198504043121437. 10.1056/NEJM198504043121437 4038784

[5] Bouleti C, Mewton N, Germain S (2015). The no-reflow phenomenon: state of the art. Arch Cardiovasc Dis.

[6] Ito H (2014). Etiology and clinical implications of microvascular dysfunction in patients with acute myocardial infarction. Int Heart J.

[7] Abbate A, Kontos MC, Biondi-Zoccai GGL (2008). No-reflow: the next challenge in treatment of ST-elevation acute myocardial infarction. Eur Heart J.

[8] Schwartz BG, Kloner RA (2012). Coronary no reflow. J Mol Cell Cardiol.

[9] Caixeta A, Lansky AJ, Mehran R, Brener SJ, Claessen B, Geńéreux P (2013). Predictors of suboptimal TIMI flow after primary angioplasty for acute myocardial infarction: results from the HORIZONS-AMI trial. EuroIntervention..

[10] Mehta RH, Harjai KJ, Cox D, Stone GW, Brodie B, Boura J (2003). Clinical and angiographic correlates and outcomes of suboptimal coronary flow in patients with acute myocardial infarction undergoing primary percutaneous coronary intervention. J Am Coll Cardiol.

[11] Ndrepepa G, Tiroch K, Keta D, Fusaro M, Seyfarth M, Pache J (2010). Predictive factors and impact of no reflow after primary percutaneous coronary intervention in patients with acute myocardial infarction. Circ Cardiovasc Interv..

[12] Thygesen K, Alpert JS, Jaffe AS, Chaitman BR, Bax JJ, Morrow DA (2019). Fourth universal definition of myocardial infarction (2018). Russian J Cardiol.

[13] Levine GN, Bates ER, Blankenship JC, Bailey SR, Bittl JA, Cercek B (2016). 2015 ACC/AHA/SCAI focused update on primary percutaneous coronary intervention for patients with ST-elevation myocardial infarctionAn update of the 2011 ACCF/AHA/SCAI guideline for percutaneous coronary intervention and the 2013 ACCF/AHA guideline for the management of ST-elevation myocardial infarction a report of the American college of cardiology/American Heart Association task force on clinical practice guidelines and the society for cardiovascular angiography and interventions. Circulation.

[14] Ding S, Pu J, Qiao ZQ, Shan P, Song W, Du Y (2010). TIMI myocardial perfusion frame count: a new method to assess myocardial perfusion and its predictive value for short-term prognosis. Catheter Cardiovasc Interv.

[15] Sianos G, Papafaklis MI, Serruys PW (2010). Angiographic thrombus burden classification in patients with ST-segment elevation myocardial infarction treated with percutaneous coronary intervention. J Invasive Cardiol.

[16] Ndrepepa G, Alger P, Kufner S, Mehilli J, Schömig A, Kastrati A (2012). ST-segment resolution after primary percutaneous coronary intervention in patients with acute ST-segment elevation myocardial infarction. Cardiol J.

[17] Kirma C, Izgi A, Dundar C, Tanalp AC, Oduncu V, Soe MA (2008). Clinical and procedural predictors of no-reflow phenomenon after primary percutaneous coronary interventions - experience at a single center. Circ J.

[18] Bouleti C, Mathivet T, Serfaty JM, Vignolles N, Berland E, Monnot C (2015). Angiopoietin-like 4 serum levels on admission for acute myocardial infarction are associated with no-reflow. Int J Cardiol.

[19] Parodi G, Valenti R, Carrabba N, Memisha G, Moschi G, Migliorini A (2006). Long-term prognostic implications of nonoptimal primary angioplasty for acute myocardial infarction. Catheter Cardiovasc Interv.

[20] Park JS, Cha KS, Shin D, Lee DS, Lee HW, Oh JH (2015). Prognostic significance of presenting blood pressure in patients with ST-elevation myocardial infarction undergoing percutaneous coronary intervention. Am J Hypertens.

[21] Singh M, Mathew V, Garratt KN, Berger PB, Grill DE, Bell MR (2000). Effect of age on the outcome of angioplasty for acute myocardial infarction among patients treated at the Mayo Clinic. Am J Med.

[22] Jaffe R, Dick A, Strauss BH (2010). Prevention and treatment of microvascular obstruction-related myocardial injury and coronary no-reflow following percutaneous coronary intervention: a systematic approach. JACC Cardiovasc Interv.

[23] Heusch G, Skyschally A, Kleinbongard P (2018). Coronary microembolization and microvascular dysfunction. Int J Cardiol.

[24] De Luca G, Suryapranata H, Zijlstra F, Van’t Hof AWJ, Hoorntje JCA, Gosselink ATM (2003). Symptom-onset-to-balloon time and mortality in patients with acute myocardial infarction treated by primary angioplasty. J Am Coll Cardiol.

[25] Cannon CP, Gibson CM, Lambrew CT, Shoultz DA, Levy D, French WJ (2000). Relationship of symptom-onset-to-balloon time and door-to-balloon time with mortality in patients undergoing angioplasty for acute myocardial infarction. J Am Med Assoc.

[26] Bae JH, Kwon TG, Hyun DW, Rihal CS, Lerman A (2008). Predictors of slow flow during primary percutaneous coronary intervention: an intravascular ultrasound-virtual histology study. Heart..

[27] Niccoli G, Burzotta F, Galiuto L, Crea F (2009). Myocardial no-reflow in humans. J Am Coll Cardiol.

[28] Prati F, Capodanno D, Pawlowski T, Ramazzotti V, Albertucci M, La Manna A (2010). Local delivery versus intracoronary infusion of abciximab in patients with acute coronary syndromes. JACC Cardiovasc Interv..

[29] Dudek D, Dziewierz A, Brener SJ, Abizaid A, Merkely B, Costa RA (2015). Mesh-covered embolic protection stent implantation in ST-segment-elevation myocardial infarction: final 1-year clinical and angiographic results from the MGUARD for acute ST elevation reperfusion trial. Circ Cardiovasc Interv.

[30] Isaaz K, Robin C, Cerisier A, Lamaud M, Richard L, Da Costa A (2006). A new approach of primary angioplasty for ST-elevation acute myocardial infarction based on minimalist immediate mechanical intervention. Coron Artery Dis.

[31] Caputo RP, Goel A, Pencina M, Cohen DJ, Kleiman NS, Yen CH (2012). Impact of drug eluting stent length on outcomes of percutaneous coronary intervention (from the EVENT registry). Am J Cardiol.

